# Hospital and Institutional News

**Published:** 1916-12-16

**Authors:** 


### HOSPITAL AND INSTITUTIONAL NEWS.

### MEMORIAL TO MR. MELHADO.

It is satisfactory to learn that a memorial is to be completed in respect and gratitude for the thirty-seven years of work which the late Mr. Clare Melhado devoted to the Middlesex Hospital. The idea is to raise a fund to endow permanently the Bland-Sutton Institute of Pathology, and a committee has been appointed to see the project through. Among the subscriptions already promised or received is one of one thousand guineas from Mr. Edmund Davis. The plan here proposed has much to commend it, but we hope that the committee entrusted with the task of completing the memorial will soon declare how they propose to associate Mr. Melhado's name with it. There could be no better memorial than one which extends the hospital's usefulness, but at the same time there must be a permanent and outward sign of the affection in which Mr. Melhado's name is held if the fund is also to be a memorial to the man who has inspired it.

### THE COLOUR BAR AT A LONDON HOSPITAL.

In a recent advertisement for resident medical officers at the Paddington Green Children's Hospital a sentence was included which has caused a good deal of resentment amongst medical men of Indian nationality, and has also elicited a protest from at least one European. The unlucky sentence in question stated that "as this is a children's hospital applications from Indian or Egyptian students cannot be entertained." It is pointed out, and justly, that the logic of this pronouncement is at fault; it is not amongst child, but amongst adult, patients that a prejudice against non-European medical attendants is apt to arise. We agree that there is no reason why Indian or Egyptian medical officers should be debarred from posts at children's hospitals any more than at those for adults. The whole question of the colour bar in hospitals is so complicated and so much overlaid by side-issues that it is unprofitable to discuss it in brief compass. Admittedly, any hospital committee which decides not to accept other than white candidates for its appointments is within its rights in so doing. Admittedly, also, if Indian and Egyptian candidates are not to be accepted, it is fairer and better in every way to word the advertisement so as to let them know that their applications are not desired. But it is distinctly unwise, in our opinion, to do what the Paddington Green authorities did—namely, to make the nature of the hospital's *clientèle* serve as an excuse for excluding non-Europeans without adequate reason or logical foundation for so doing.

### HUMANISING MECHANICAL TREATMENTS.

Sir George T. Beatson lately gave an interesting address, before the Scottish Branch of the Red Cross Society, on the treatment of disabled soldiers. In describing the manual curative workshops, one of which is being installed at Bellahouston, he pointed out that soldiers who found mechanical treatment very monotonous could make equally useful movements in other ways. Instead of squeezing a ball to exercise the muscles of the hand, shearing a hedge or clipping a cloth was equally useful and much more amusing. The plane and the saw exercise the forearm and shoulder better than any pulley, and the fret-saw on a treadle machine is more natural and as effective as pedalling a dummy bicycle against a brake. These workshops are curative and have connection with those named after Lord Roberts, the object of which is to teach disabled men a new trade. Sir George Beatson believes a big national scheme may grow from these beginnings.

### THE POWER OF THE SPECIALIST.

At a private meeting held at Hastings under the chairmanship of Sir Henry Lunn, the best means of popularising Hastings as a spa among consultants and their patients were discussed in the presence of certain local doctors. France, it was said, is to develop its own spas after the war, and Hastings, with its climate, history, and chalybeate springs, might also develop its natural resources. Sir Henry Lunn declared that for some reason medical favour was withdrawn from Hastings a few years ago. But this, he said, was virtually a question of medical etiquette. At one time a health resort could have been made or marred by the action of a few London specialists: now there were distinguished consultants in every city. The medical baths must be taken in hand and brought up to date. It was suggested that eminent consultants should be invited to the town, so that they might learn its virtues at first hand. We wish well to Hastings and our English spas generally, but it is impossible to restrain a friendly smile at the tone adopted towards consultants in some of the remarks which we have summarised.

### THE GERM OF SCARLET FEVER.

Scarlet fever is one of those disorders which, by their analogy with diseases known to be caused by microbic infection, is always assumed to be due to some as yet unidentified micro-organism. Numerous attempts have been made by research workers to identify and isolate this elusive parasite; and several times claims to success have been put forward, only to be dispelled by further investigation. The latest efforts in this direction, at any rate in this country, have been those of Dr. Mair, research pathologist to the Metropolitan Asylums Board, who has recently elaborated a previous account, published last year, of the *Diplococcus scarlatinæ*, which he believes to be the causal organism of this disease. This diplococcus resembles closely the diplococcus of pneumonia; and, like that organism and the *Bacillus diphtheriæ*, it is found in normal fauces as well as in those of patients suffering from scarlet fever. It has not hitherto been shown to produce in monkeys definite nephritis, similar to the acute nephritis so commonly seen in mankind as a result of scarlet fever, but it does produce in those animals a "scarlatinal rheumatism" which is thought to present analogies with that of human beings. For mice, rats, rabbits, and guinea-pigs its toxicity appears to be of a low order, which has probably hampered the prosecution of Dr. Mair's researches. After reviewing the evidence for and against the claims put forward by the latter, the *Medical Officer* "cannot yet accept the case as proven on the evidence he has adduced."

### GENERAL MEDICAL COUNCIL PROCEEDINGS.

Routine work continues even in times of war and of dearth of medical workers, as we are reminded by the issue of reports of the agenda (or rather *acta*) at recent meetings of the above body. The Sub-Commmittee for Dental Education and Examination have been in communication with professional bodies in New South Wales, Victoria, and South Australia, having exchanged information with these as to the conditions governing dental registration in the respective Colonies. A dental graduate of the University of Kieff was admitted to the Register of Foreign Dentists. The Branch Council for England considered an application from an Havana medical practitioner, qualified in England and at Paris, for admission to the Medical Register, which was granted. The remaining business is reported in General Council minutes of last month, and concerned disciplinary inquiries, in one or two cases at the instance of National Insurance Committees. Six medical men, two of whom qualified from Edinburgh, two from Glasgow, one from London, and one from Manchester, were arraigned on various charges, the least serious of which were two of "covering" uncertified midwives. Every practitioner save one was represented by counsel. One charge out of the six was deemed not proved; while in the remaining five cases warnings were given to the defendants, conditional upon good conduct for specified future terms. None of them suffered the penalty of having his name erased from the Register.

### AN EXETER SMALL-POX HOSPITAL SCHEME.

At a recent meeting of the Exeter City Council strong opposition was manifested to the proposals of the Sanitary Committee in respect of a new small-pox hospital which the Council has had in contemplation for some time. It appeared that an application to the Local Government Board for sanction of a loan to meet the expense of the building has been refused; and the Board have also further refused a request for an official inquiry as to the suitability of the site and similar matters. The Sanitary Committee discovered that they could defeat this obstructive attitude on the part of the Local Government Board by building the hospital piecemeal out of revenue, and recommended that plans should be drawn up for carrying out this ingenious idea. After an animated debate, in which differences of opinion with regard to questions of fact were expressed, the dissentients on the Council succeeded by a considerable majority in referring back the recommendations of the Sanitary Committee. A good deal of criticism of the methods of this committee was expressed with some freedom; and the upshot of a debate, the report of which is somewhat difficult to follow, is not in doubt—namely, a rebuff for the scheme of a new small-pox hospital at the present time.

### NOTTINGHAM HOSPITAL AND STATE CONTROL.

The governors of the Nottingham General Hospital are opposed to any movement in the direction of State control. They believe that there are voluntary resources still untapped, and in this connection are appealing to munition workers, who are receiving greatly increased wages, to support liberally the institution. The Government has sought the assistance of the governors in the campaign against venereal diseases, and this will be readily given provided the independence of the hospital is fully safeguarded. Though no indication has been given of a disposition to extend State control to the Nottingham Hospital and similar institutions, the governors feel it desirable to emphasise their position in the matter, in view of any State assistance which may be given in dealing with venereal diseases.

### MUNICIPAL GIFT TO COVENTRY HOSPITAL.

The Coventry City Council has unanimously decided to give a special donation of £1,000 to the Coventry and Warwickshire Hospital as a contribution to the additional expenditure incurred in the admission and treatment of wounded soldiers. The Mayor, Councillor A. S. Hill, moved, Alderman Drinkwater seconded, and Dr. Soden supported the proposal. The gift is worthy of the Corporation and of the cause to which it will be applied. We hope that other municipalities will take the example of Coventry to heart, and that many gifts of a similar kind will be made in other parts of the country.

### MORE HOSPITAL TROUBLES IN NEW SOUTH WALES.

The New South Wales Branch of the British Medical Association has lately had under consideration an action of the Government of that Colony which amounts, in the opinion of the doctors concerned, to one more attempt to exploit the medical profession, and is to be strenuously resisted. The State contributes considerable subsidies to the voluntary hospitals in that Colony; and, by an order of the Ministry of Public Health, henceforth all Government servants injured while in the execution of their duty are to receive free treatment in hospital, as one of the conditions attaching to the Government grant. At first sight this may appear reasonable; for doubtless the contribution from the Government is many times in excess of any expense the hospitals are likely to incur in treating such servants of the Government as may be injured while on duty. The order, however, quite fails to take into account the fact that the (unpaid) medical and surgical staffs of these hospitals finger not one penny of the Government grants, and give their services free to the hospitals because the latter are charitable institutions. They do not, therefore, see any obligation to attend on such terms persons who may be perfectly well able to pay for their own medical attendance; and have passed a resolution reaffirming the "principle of refusing, except in cases of urgency, to be exploited by persons who are in a position to pay for our services, and we cannot differentiate between employees of the Government and others." It would surely have been better if the Minister had invited the opinion of the parties concerned (the hospital staffs and the hospital managers) before he issued the order which has caused so much heart-burning.

### ZEPPELINS AND A POOR-LAW PROBLEM.

The latest critic of the Government insurance scheme against damage by hostile aircraft and bombardment is the Association of Poor-Law Unions in England. It previously refrained from joining the movement of protest for fear of trespassing beyond its province; but now that some of those whose homes have been damaged or destroyed have come to their Boards of Guardians for relief, including, we understand, persons normally outside the range of the Poor Law, the Association feels entitled to express its criticism with that of other bodies.

### AN EXTRAORDINARY SOURCE OF INCOME.

It has been currently reported in the press that the Bolton, Lancashire, Tribunal allowed a conscientious objector, who asked for a renewal of his exemption certificate, to gain his request on condition that he paid five shillings a week to the Bolton Infirmary out of the difference between his Army pay and his present earnings as a brushmaker. It would be interesting to know whether such a decision is allowed by law: whether legal or not, such a fine is a very strange source of income for a hospital.

### THE "FUTURE DECLINE" OF MEDICAL PRACTICE.

Prophecies concerning the future of medicine and hospitals have some interest when they are made by medical men or institutional managers. But what will be thought of a recent prophecy to the effect that hospital work will greatly increase and private medical practice diminish accordingly? It is true that the School Medical Service, and, indeed, the public Medical Service generally, deserves and will probably receive considerable extension after the war. It is also true that we may expect a larger number of hospital beds and smaller waiting lists when the time comes for the diminution of military patients. But these facts hardly touch the fringe of private practice. The very poor have always had their club doctors. The middle and upper classes have always preferred medical attention in their own homes except in the case of operations. These last will have to be performed, as in the past, in nursing homes, or preferably in pay wards attached to voluntary hospitals. How, then, can the prophesied decline in private practice occur after the war? Will not the mainstay of private practice then as now, apart from surgery, concern cases which can be treated most suitably in the home of the patient or in the consulting-room of the physician? We leave the verdict to the common sense of our readers.

### THE DAVID LEWIS COLONY REPORT.

A record of steady progress was unfolded at the annual meeting of the governors and subscribers of the David Lewis Manchester Epileptic Colony. The managing committee stated that the daily average number of the colonists at Warford was 318. The farm has yielded a profit of £420 and the poultry farm £60. The colonists have taken a great interest in the making of garments for wounded soldiers, and up to the end of August 100 suits of pyjamas were distributed among the military hospitals in Manchester and district. A few former colonists in whom the disease had been arrested joined the Army, and were reported to be standing the strain well. Many of the patients at the colony showed a desire to enlist, but few were suitable for the Army. Lord Sheffield, who presided at the meeting, mentioned that Dr. Hopkinson, chairman of the managing committee, is away in India, but he said that so long as Dr. Alan McDougall remained at the colony they could rest satisfied that everything would be carried on in the best possible way.

### BOARDING-OUT OF MEDICAL STAFF.

There is a shortage at Lambeth Infirmary of accommodation for the assistant medical officers, and the medical superintendent, Dr. Lionel Baly, suggested that one of the officers should make arrangements for his own board and residence. After considerable difficulty he had succeeded in securing suitable apartments at the rate of three guineas a week, which he asked the board to pay. The infirmary visiting committee, however, considered the amount asked for excessive, and on their recommendation the board decided that it be reduced to two guineas a week. The matter received due consideration at the meeting of the Guardians, the majority of whom felt that the smaller amount was adequate. They overlooked the fact that the infirmary is situated in a district where it is extremely difficult to obtain suitable accommodation for a professional man, and it was only by appealing to someone in the same sphere of life as himself that the doctor was able to get the accommodation he desired. In these circumstances the Guardians might have stretched a point, especially when the high price of commodities are taken into account.

### THE BIRTH OF TUBERCULOSIS AFTER-CARE ASSOCIATIONS.

At the present time, when a large proportion of public and private charitable funds is necessarily diverted to objects directly connected with war work, it is becoming increasingly difficult to obtain the needful support for other enterprises. Never were the claims of the tuberculous so pressing as now, and yet there appears to exist in certain quarters an unwillingness to prosecute the anti-tuberculosis campaign with sufficient vigour to prevent the benefits derived from sanatorium treatment from being nullified, or, at any rate, spoiled, through neglect to follow up the cases upon their return to home conditions. This is apparent in the somewhat half-hearted manner in which after-care is provided for. In some districts nothing is done, while in others good work has been discontinued for one reason or another; and, in the meantime, the poor consumptive is left to take care of himself as best he may. It is gratifying to learn, therefore, that during the present month two after-care associations, or committees, have been formed, one for the Frome District of Somerset and the other for the Administrative County of Cambridge. At a meeting held for the inauguration of the former, Dr. Latimer J. Short, the energetic county tuberculosis officer for Somerset, instanced the fact that there were two-thirds as many deaths from tuberculosis in the first year of the war as of men killed on the battlefield. In the provision of nourishment, beds, clothing, better housing accommodation, and in a hundred other ways, a thoroughly representative after-care association composed of alert individuals can accomplish much that a county council cannot do, and is able to supplement to a high extent official action by voluntary help and supervision. It is little else than a scandal that any district should remain without a flourishing tuberculosis after-care association.

### A CENTRAL HOSTEL FOR ARMY NURSING PROBATIONERS.

The much-discussed Supply of Nurses Committee has evidently settled down to work, for it has issued an interim report to the War Office recommending the establishment of a central hostel where young women anxious to undertake nursing work under a contract during the war, but without hospital experience, can live, receiving lectures in the hostel, and, if possible, attending for daily work as probationers in civil hospitals and infirmaries. The proposal has received the favourable consideration of the War Office, who hope, with the co-operation of the British Red Cross Society, to arrange the details of the scheme at an early date. It may be noted, in connection with the dearth of nurses which this scheme connotes, that the Queen Victoria's Jubilee Institute for Nurses is also heavily depleted by the demands of the war. So many of the "Queen's Nurses" have left their posts under the Institute to nurse the wounded that the work in the districts has been necessarily interfered with. At the same time there has been an increasing demand for district nurses in connection with the development of public health work.

### INCREASED SALARIES FOR NURSES.

The Secretary of the War Office has announced that it has been decided to increase the pay of all members of Queen Alexandra's Imperial Military Nursing Service Reserve and the Territorial Force Nursing Service who, having completed twelve months' service, are willing to sign an undertaking for general Army service as long as required. The pay of V.A.D. and special probationers in military hospitals staffed by Q.A.I.M.N. Service or the T.F.N. Service will also, after they have completed six months' approved service, be raised from £20 to £22 10s. per annum; and, provided they are willing to sign an undertaking for general Army service for as long as necessary, they will be eligible for further half-yearly increments of £2 10s. up to a maximum of £30 a year. There is nothing unfair in these regulations; for those who sign the undertaking to stay on until the end of the war are mortgaging the future to a much greater degree than those who sign only for a limited period, and deserve higher pay in consequence. Besides these conditional increases of pay, the rates of allowance for board and washing for all nurses and V.A.D. probationers serving with the Army have been increased by four shillings a week: they are now nineteen shillings a week for home, and twenty-five shillings a week for foreign service. Nor is it in the Army alone that rates of pay for nurses are on the up grade. The London Hospital authorities have adopted a scheme of increased salaries for the nursing staff which will entail an additional cost to the management of £4,000 a year.

### A SUGGESTION FOR STATE PHARMACIES.

A dispute is in progress between the Manchester Insurance Committee and the panel doctors on the vexed question of prescribing. Mr. Davies, the chairman of the committee, contends that there are no limitations to prescribing for insured patients. This statement is controverted by the doctors, and Dr. A. W. Martin, the Medical Officer for Gorton, points out that in one year he was surcharged £30 because he treated insured people like his own private patients. He claims that the only solution of the problem is to establish national pharmacies, where patients would get their prescriptions dispensed by State-paid chemists at a much lower cost than at present.

### THIS WEEK'S DRUG MARKET.

It need hardly be said that Germany's peace kite has not brought down the prices of drugs. This in itself suffices to show what operators in the drug market think of the proposal, for we may be sure that were there any sound reason to believe that peace was really in sight the values of many drugs would come down almost like the proverbial rocket stick. As a matter of fact values are well maintained, and in several cases the upward tendency continues strong, notwithstanding that we are approaching the quietest season of the year. Camphor is again dearer. Balsam copaiba is very scarce, and business has been done at high prices. Scammony root is almost unobtainable, and scammony resin is also very scarce. Extravagant prices have to be paid for any small lots of resorcin that are available. Chrysophanic acid is very scarce. Potassium permanganate is still advancing in price. Valerianates are in very short supply owing to the scarcity of valerianic acid. Stocks of cocaine are still extremely low, and the price has an upward tendency. Acetylsalicylic acid still has an easier tendency, but salicylate of soda is for the moment somewhat steadier. Salol is again slightly lower in price

